# Low-threshold secondary and tertiary suicide prevention measures for individuals with suicid-related thoughts and behaviors: a scoping review

**DOI:** 10.3389/fpubh.2026.1868906

**Published:** 2026-09-28

**Authors:** Cora Spahn, Lena Spangenberg, Clara Sturbeck, Doreen Hoffmann, Heide Glaesmer

**Affiliations:** Department of Medical Psychology and Medical Sociology, Leipzig University, Leipzig, Germany

**Keywords:** lifeline, low-threshold, reduction STB, scoping review, secondary, suicide prevention, tertiary

## Abstract

**Introduction:**

Suicide rates remain persistently high in many regions worldwide, including Germany. The 2024 German National Suicide Prevention Strategy emphasizes the strengthening of evidence-based approaches. In this context, a comprehensive synthesis of low-threshold secondary and tertiary suicide prevention measures for adults is required.

**Methods:**

This scoping review set out to address this need and followed established methodological guidance and the PRISMA-ScR reporting guidelines. PubMed and Web of Science were searched on September 1, 2025, for studies published from January 1, 2005, onwards. To be eligible for inclusion, studies needed to examine low-threshold secondary or tertiary suicide prevention measures targeting adults with suicide-related thoughts and behaviors (STBs). Interventions focusing on primary medical care, psychotherapy, pharmacotherapy, gatekeepers and means restriction were excluded.

**Results:**

Out of 1,897 records, 24 studies met the inclusion criteria. Most studies investigated voice-based (*n* = 9; 37.5%) and text-based (*n* = 7; 29.2%) crisis interventions within suicide prevention lifelines. These interventions consistently demonstrated reductions in suicidal ideation and psychological distress, with one exception: a moderated peer-to-peer forum. Outreach messages (*n* = 3; 12.5%) and web-based educational (and counseling) measures (*n* = 5; 20.8%) showed promising but more heterogeneous findings. Outcomes indicated potential benefits, though the evidence was less consistent.

**Discussion:**

The findings suggest that voice- and text-based crisis interventions are supported by a growing evidence base, primarily demonstrating short-term effects. Web-based educational (and counseling) measures and outreach messages, while heterogeneous, show potential for individuals with STBs through the use of emerging technologies. Future research should examine mechanisms of action, long-term outcomes, and employ randomized controlled trial designs (where feasible) to strengthen the evidence base.

**Systematic review registration:**

https://doi.org/10.17605/OSF.IO/K2B83.

## Introduction

Suicid-related thoughts and behaviors (STBs) constitute a major global public health concern (1). Over the past decades, various suicide prevention initiatives, improved access to care, and societal developments have contributed to a notable decline in suicide rates in many countries (1, 2). Nevertheless, this decline has not progressed in recent years to the extent anticipated under the WHO Sustainable Development Goals (SDGs) (3). In Germany, following a marked reduction between 1980 and 2000, suicide rates have remained largely stable over the past 25 years, ranging between 9,000 and 10,000 cases annually (2). In 2024, the German Federal Ministry of Health published a National Suicide Prevention Strategy, with the aim of strengthening evidence-based suicide prevention in Germany (4). As part of this initiative, recommendations are needed that specifiy which secondary and tertiary preventive measures are most suitable to support the strategy. Against this backdrop, the present scoping review was conducted to synthesize the available evidence on low-threshold secondary and tertiary suicide prevention measures, to identify effective measures, and to provide evidence for the German National Suicide Prevention Strategy.

In the final report of the German National Suicide Prevention Programme, Fiedler et al. (5) define low-threshold suicide prevention as encompassing “professional, semi-professional, and lay-supported services” that provide “barrier-free access for help-seeking individuals across the full spectrum of suicidal ideation and behaviour” and aim to reduce suicidality while providing psychosocial support. According to the authors, such services should be easily accessible, confidential, ideally free of charge, and available without waiting periods.

Secondary and tertiary suicide prevention measures differ from primary prevention in that they target individuals who already experience or have experienced STBs. Primary prevention, in contrast, focuses on the general population or at-risk groups without manifest symptoms, aiming to prevent the emergence of STBs. Secondary measures relate to the early management of suicidal ideation, for example through crisis intervention, whereas tertiary measures focus on relapse prevention, such as the prevention of repeating suicide attempts (6). In practice and research, secondary and tertiary measures are often difficult to distinguish and are therefore considered jointly in this review (7). Table 1 provides an overview of (low-threshold) measures for primary, secondary, and tertiary suicide prevention.

**Table 1:** Overview of primary, secondary, and tertiary suicide prevention measures.

Aspect	Primary suicide prevention	Second-, and tertiary suicide prevention
Target population	General population and individuals at increased risk	Individuals with current or past STBs
(Low-threshold) measures e.g.	Public awareness campaigns; school- and community-based education; responsible media reporting guidelines; means restriction	Crisis hotlines/Lifelines; text-based (chats) crisis intervention; psychosocial interventions; gatekeeper programs

In practice, a wide range of suicide prevention measures are implemented, and a variety of secondary and tertiary suicide prevention strategies have been analyzed in the research literature. Over the past 10–15 years, research on these measures has increased markedly. Several scoping and systematic reviews have attempted to provide an overview of the various areas of suicide prevention and the available evidence. Some reviews have focused on measures for specific populations [e.g., men (8, 9), students (10), older adults (11)], or geographic regions [e.g., rural areas (12)], while others have examined specific intervention types, such as web-based suicide prevention (13–15).

Miller et al. (16) primarily examined the mechanisms underlying the effectiveness of Lifelines in the United States. The focus of Miller et al. (16) is not primarily on the effects of the intervention on STBs, and their analysis is limited to a single region. A recent review published in 2025 assessed the effectiveness of gatekeeper programs (17). Among other outcomes, changes in the behavior of trained individuals were observed (e.g., whether they intervened more frequently or in different ways).

In summary, existing reviews have largely focused on specific interventions, target populations, or geographic regions, or have addressed aspects other than the effectiveness of interventions in relation to STBs or psychological distress. To date, no comprehensive overview exists regarding the effects of low-threshold secondary and tertiary suicide prevention measures on STBs in adults. This gap is addressed by the present review. Where previous research intersects with our research question, it has been taken into account. For this reason, gatekeeper programs will not be addressed in our review.

Excluded from the present review are also suicide prevention measures with a main focus on medical care (e.g., outpatient and inpatient care), pharmacotherapy, and psychotherapeutic treatment, as strong evidence already exists in these domains. Some reviews covering multiple suicide prevention strategies have demonstrated effectiveness primarily for these measures. Mann et al. (18) showed that primary care training, psychiatric treatment and follow-up, as well as pharmacotherapy, are effective in reducing STBs. Additional studies provide comprehensive evidence for psychotherapeutic interventions (19–22). These measures are also reflected in clinical recommendations, including those of the National Institute for Health and Care Excellence (23). A particularly suicide-focused psychotherapy appears to be a potentially effective approach (24).

Zalsmann et al. (25) reviewed evidence for seven types of suicide prevention measures (public and physician education, media strategies, screening, restriction of access to means, treatment, and internet or hotline support) and also found support for pharmacological and psychotherapeutic interventions, school-based awareness programs, and means restriction. For the remaining types of measures, the need for future research was emphasized due to the lack of randomized controlled trials (RCTs). Means restriction is not considered in the present review, as robust evidence already exists and it is classified as structural prevention because it modifies environmental conditions rather than individual behavior [e.g., (26, 27)].

The aim of this scoping review is to systematically identify and map the existing evidence on low-threshold secondary and tertiary suicide prevention measures for adults. After excluding primary medical, psychotherapeutic, pharmacological interventions, and gatekeeper programs, examples of measures relevant for our aims and based on Mann et al. (18, 25) and Zalsmann et al. (25)-include Lifelines (voice- or text-based crisis support), on site counseling, information and training programs, risk detection (screening) and outreach, as well as self-help and peer-to-peer support. The broad research question (Which low-threshold prevention measures targeting adults can be considered effective based on current available evidence?), heterogeneous measures, and assumed methodological diversity of the studies in the field (ranging supposedly from RCTs, quasi-experimental to qualitative studies) suggest that a scoping review is the most appropriate approach (28).

## Methods

This scoping review followed the approach outlined by Arksey et al. (28), more recent adaptations (29–32) and the PRISMA-ScR guidelines (33). The following steps were carried out: (1) Development of a research question, (2) Identification of relevant studies, (3) Selection of studies, (4) Structured data extraction from the included studies, (5) Synthesis and reporting of results. The study was preregistered on the Open Science Framework (34).

### Search strategy

To identify relevant studies, searches were conducted in two databases: PubMed and Web of Science. The search strategy was developed through an iterative process. Three content domains were defined (suicide prevention; secondary/tertiary suicide prevention measures; access/low threshold), each represented by various search terms linked with the Boolean operator AND. Within the three content domains, different search terms were linked with the Boolean operator OR. A requirement was that suicid^*^ and prevention^*^ appear together in the title and abstract; the remaining search terms could appear in either the title or the abstract. Appropriate MeSH terms were used in the PubMed search. The full search strategies and database-specific adaptations are available on OSF (https://osf.io/kjt39) and in the Supplementary material. An example of an abbreviated PubMed search query is (suicid^*^[ti] AND prevention^*^[ti]) AND (suicid^*^[tiab] AND prevention^*^[tiab]) AND (“Suicide Prevention”[Mesh] OR “Crisis Intervention”[Mesh] OR helpline^*^[tiab] OR “Counseling”[Mesh] AND “Health Services Accessibility”[Mesh] OR barriers[tiab] OR “health care barrier^*^”[tiab] OR “low-threshold”[tiab]). In addition to database searches, the reference lists of included articles were manually screened.

The search was restricted to articles published between January 1, 2005, and September 1, 2025 (the date of the search). This time period was applied because it encompasses the emergence and major developments of suicide prevention interventions and related research. Only articles published in English or German were considered.

### Inclusion and exclusion criteria

The inclusion and exclusion criteria were developed through an iterative process. Studies were included if they assessed low-threshold secondary or tertiary suicide prevention measures. Secondary and tertiary suicide prevention encompasses measures for individuals with current or past suicidal thoughts and behaviors (STBs). To clearly classify an intervention as secondary or tertiary suicide prevention, individuals with STBs were required to participate in the intervention and to be involved in its evaluation. In addition, studies had to assess at least one outcome related to STBs or psychological distress.

An additional inclusion criterion was low-threshold access. Accordingly, only studies on interventions that were accessible without major formal barriers (e.g., without referral requirements, long waiting times, or financial barriers, or with anonymous access) were included. Furthermore, only studies involving individuals aged 18 years and older were considered. Only peer-reviewed articles were included, encompassing all study designs, such as randomized controlled trials (RCTs), observational studies, and quantitative and qualitative research.

As described in the Introduction, studies on suicide prevention measures primarily focused on medical care (e.g., outpatient and inpatient care and related aftercare interventions), pharmacotherapy, psychotherapeutic treatment, or means restriction were excluded from the present review. During the review process, the eligibility criteria were further refined to exclude gatekeeper programs, as a dedicated review on this topic had recently been published (17). No search of gray literature was conducted.

Since critical appraisal of methodological quality is not a required component of scoping reviews (28), no formal quality assessment was conducted or used to inform decisions regarding study inclusion or exclusion. Methodological limitations of the included studies are presented in the Supplementary material accompanying the extracted data (34).

### Study selection

All records retrieved from the databases were imported into the web-based tool Rayyan (www.rayyan.ai), where duplicates were removed by CS2. The initial title and abstract screening, as well as the management of included and excluded articles, was also conducted using Rayyan. The entire study selection process was carried out independently by CS1 and CS2. In cases of disagreement, HG, LS and DH were consulted, and a consensus was reached through discussion. First, titles and abstracts of all articles were screened, and a preliminary selection was made based on the inclusion and exclusion criteria. Of the pre-selected studies, all full texts were reviewed to determine the extent to which the inclusion and exclusion criteria were met. Disagreements were discussed jointly by CS1, CS2, HG, LS and DH to decide for or against inclusion. For example, it was discussed whether services for individuals bereaved by suicide without STBs should be considered tertiary prevention, and to what extent identification based on suicide-related Google searches adequately captures individuals experiencing STBs. In both cases, the studies were not included because individuals with STBs were not clearly involved. Figure 1 shows the detailed study selection process.

**Figure 1:**
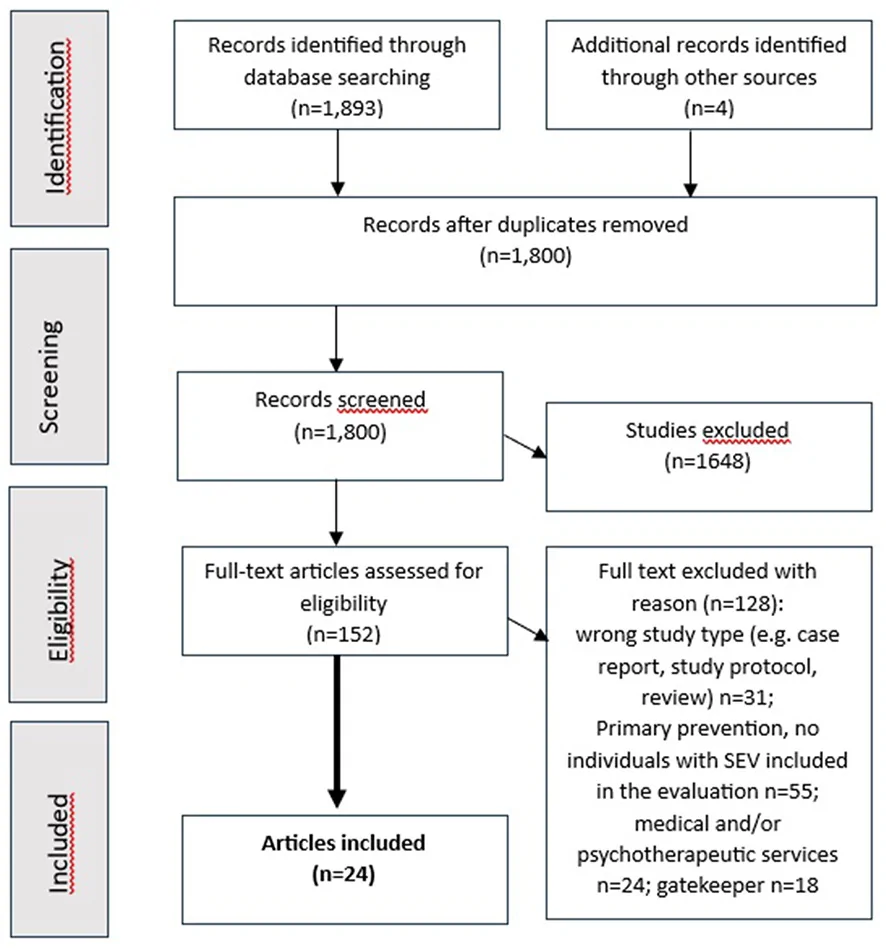
Flowchart of the study process.

### Data extraction, charting, data synthesis

In accordance with the procedure for scoping reviews (28, 31), the following data were extracted from the included studies and compiled in an Excel spreadsheet (34): authors, year of publication, country, study design, population including number of participants, methods, outcome, title, and DOI. Data preparation was carried out by CS1 and CS2. Together with HG and LS, the extracted data were summarized and synthesized according to several aspects, including the examination of specific risk groups, the description of measures/interventions, and the effectiveness of the measures. The data synthesis was based on a thematic analysis, through which clusters were formed according to the type of intervention.

## Results

A total of 1,893 articles were identified in the PubMed and Web of Science databases using the predefined search terms. Additionally, four articles were identified through hand-searching the reference lists of the included studies. After the duplicates were removed, 1,800 items remained. Of these articles, 1,648 were excluded by screening the titles and abstracts (for example, in cases where no evaluation was conducted, where the measure was primarily preventive, or where the target population consisted of individuals under 18 years of age). A total of 152 articles underwent full-text screening; in this step, a further 128 articles were excluded, and 24 articles were included in the scoping review analysis. Figure 1 shows the flowchart of the study selection process and the reasons for exclusion at the full-text screening stage.

### Description of the studies

Table 2 provides an overview of the 24 selected studies and their characteristics. All studies on low-threshold secondary or tertiary suicide prevention services included individuals with STBs in their evaluations. Some of the studies (35–37) also include individuals without STBs in their evaluations; in these cases, the analysis focused specifically on the effects on individuals with STBs. Of the studies, 37.5% (*n* = 9) examine the effects of voice-based crisis intervention via telephone counseling (Lifeline). These studies were published between 2012 and 2025. The sample sizes investigated range from *n* = 300 to *n* = 4,657.

**Table 2:** Overview on included studies (*n* = 24).

References	Country of origin	Study design	Population	Research aim	Setting/measures	Outcome
Voice-based crisis intervention						
Gould et al. (36)	USA	Quantitative study	*n* = 376 (Callers to lifeline)	Assessing post-call mental health care utilization and barriers following Lifeline calls.	National Suicide Prevention Lifeline, telephone interviews.	44% of referred suicidal/crisis callers used mental health care; health insurance (especially among crisis callers) and being in treatment at the time of the call were associated with higher utilization; main barriers: attitudinal, financial, and structural.
Gould et al. (39)	USA	Quantitative/ intervention study	*n* = 1,507 (Monitored calls to lifeline)	Evaluating the impact of implementing ASIST across the lifeline network.	Lifeline centers, monitored calls, caller self-ratings, and end-of-call states.	Calls with ASIST-trained counselors showed greater end-of-call improvements (less depressed, suicidal, and overwhelmed; more hopeful).
Gould et al. (40)	USA	Quantitative study	*n* = 550 (Callers to lifeline, follow-up clients)	Evaluating follow-up calls to suicidal lifeline callers and perceived effectiveness.	Lifeline follow-up, counselor questionnaire, client telephone interview.	Reporting that 79% of clients said the intervention stopped them from suicide and kept them safe (90%); showing that follow-up calls to suicidal individuals can reduce perceived risk of future suicidal behavior.
Britton et al. (41)	USA	Quantitative/ intervention study	*n* = 647 (Callers to lifeline)	Evaluating immediate outcomes of veterans crisis line calls (distress, suicidal ideation, suicidal urgency).	VCL telephone calls; independent coders rated first and last call segments (distress, suicidal ideation, and urgency).	Significant improvements during calls, with distress, suicidal ideation, and suicidal urgency decreasing from call start to call end.
Pauwels et al. (42)	Belgium	Quantitative/ intervention study	*n* = 487 (Callers to lifeline)	Testing whether helpline contact reduces crisis/suicidality indicators.	Suicide prevention helpline, automated pre- and post-call survey.	Significant pre–post improvements on crisis/suicidality-related indicators (callers reported reduced crisis/suicidality after the call).
Coveney et al. (43)	UK	Quantitative/ intervention study	*n* = 1,309 Respondents (Callers to lifeline)	Describing caller characteristics and reasons for contact; evaluating the service received.	Samaritans helpline; online survey.	High self-reported satisfaction; contact perceived as helpful; callers reported feeling less suicidal, lonely, afraid, and anxious.
Shaw et al. (44)	Taiwan	Quantitative/ intervention study	n=63,696 (Callers to Lifeline)	Describing caller profiles and testing whether the hotline reduces distress/suicide risk during the call.	Taiwan NSPH, hotline records, within-call change in emotional distress/suicidality.	Significant decreases in emotional distress and suicidality/suicidal urgency during calls (across all caller groups).
Zeinoun et al. (38)	Lebanon	Quantitative/ intervention study	n=4,657 (Callers to Lifeline)	Evaluating immediate distress reduction and predictors of distress.	Lebanon's National Emotional Support and Suicide Prevention Helpline, anonymous call data, distress ratings pre/post.	Large pre-post reductions in subjective distress from call start to end.
Mishara et al. (45)	Canada	Quantitative/ intervention study	*n* = 1,206 (Callers to lifeline)	Comparing the effectiveness of telephone suicide prevention help provided by volunteers vs. paid staff.	Silent monitoring, measures: helper response, suicide urgency, and the Crisis Call Outcome Rating Scale (CCORS); 1-week follow-up.	No significant differences in outcome measures (suicidal urgency, symptomatology, depression, contracts, follow-up behavior).
Text-based crisis intervention						
Mokkenstorm et al. (46)	Netherlands	Mixed methods	*n* = 106 (Forum users completing the survey)	Identifying the reach, benefits, and potential harms of an online forum embedded in a suicide prevention platform.	113 participants in the suicide prevention online forum; a cross-sectional web questionnaire (including SIDAS); open-ended questions about benefits and shortcomings; content categorization of moderated posts.	Low reach; mixed immediate effects (53% reported no change, 35% felt better, 12% felt worse); risks included 12% visiting to find suicide methods and 3% to find a suicide partner; overall questionable benefit–risk ratio.
Côté et al. (47)	Canada	Quantitative content analysis	*n* = 112 (Users of Text-counseling)	Describing users and perceived impact, and identifying text intervention characteristics associated with positive and negative effects.	Canadian Suicide Prevention Service (CSPS) text helpline; transcript-based content analysis of suicide-related text exchanges; counsellor assessments; pre-call questionnaire; CCORS (Crisis Call Outcome Rating Scale).	Distress decreased significantly from beginning to end of text exchange; more words exchanged were linked to more thorough exploration and action planning and to more positive CCORS items.
Côté et al. (48)	Canada	Quantitative/ intervention study	*n* = 259 (Users of text-counseling)	Evaluating the effectiveness of a centralized digital suicide-prevention strategy platform (reach, identification, intervention).	Suicide.ca platform; data from google analytics, firebase, and CRM; self-assessment questionnaires (e.g., SIDAS-FR); triage questionnaire; counsellor reports; post-intervention survey.	The triage personal-information item on the platform led to high dropout rates; chat users showed higher suicide risk than self-assessment users; counselors reported positive impact in 56% of contacts; users reported satisfaction and positive outcomes in the post-intervention survey.
Côté et al. (49)	Canada	Quantitative content analysis	*n* = 146 (Users of text-counseling)	Describing users and perceived impact, and linking intervention practices to better outcomes.	CSPS textline; SIDAS; pre- and post-emotion and coping items.	78% reported being less upset, with a significant decrease in distress; 54% reported increased ability to cope; 77–79% rated the session as helpful.
Gould et al. (50)	Canada	Quantitative/ intervention study	n=13,130 (Users of Text-counseling completing the survey)	Assessing the effectiveness of Lifeline Crisis Chat interventions and correlates of perceived effectiveness.	National Suicide Prevention Lifeline Crisis Chat (LCC); pre- and post-chat survey (5-point scale); suicidal thoughts; perceived helpfulness; post-chat emotional change (hopeful, depressed, overwhelmed, suicidal); counselor engagement.	Distress significantly reduced from chat start to end; among suicidal chatters, two-thirds found the chat helpful, and 45% reported being less suicidal by chat end.
Janssen et al. (51)	Netherlands	Quasi-experimental study	n=756 (Users of Text-counseling)	Testing whether Motivatinal Interviewing (MI) training improves chat-helpline outcomes compared with treatment as usual.	113 participants in the Suicide Prevention chat; pre- and post-chat questionnaires; MI-Scope to code counselor behavior and visitor reactions.	Both conditions showed pre-post improvements in suicidal ideation and psychological risk factors; no significant differences between MI and TAU.
Mokkenstorm et al. (52)	Netherlands	Quantitative/ intervention study	*n* = 526 (Chat logs)	Evaluating the reach and outcomes of crisis chat and comparing them with telephone hotline findings.	113 Online crisis chat; chat-log ratings; crisis and suicidality states; helper behaviors.	Outcomes comparable to telephone hotlines with overall positive effects; suggesting a stronger focus on suicidality during chats and the development of best practices specific to online crisis intervention.
Outreach messages in web-based contexts						
Liu et al. (53)	China	Feasibility study	*n* = 12,486 (Social media posts/users)	Testing the feasibility and acceptability of proactive suicide prevention combining Machine Learning (ML)-based detection with crisis management via social media.	Sina Weibo (Chinese microblogging platform); ML to detect suicidal-content comments; direct message outreach with questionnaires; counselor support via DM; pre- and post-text analysis (death-related vs future-oriented words).	ML detection performed well; substantial engagement; 90% of respondents reported suicidal ideation; 52% had not sought help; acceptability was 69%; post-consultation language showed reduced death-oriented and increased future-oriented word use.
Keaser et al. (54)	Israel	Qualitative study/thematic analysis	*n* = 116 (Responses from identified users)	Exploring users' perceptions of volunteer-delivered social media outreach and its potential to promote help-seeking.	Suicide prevention outreach on social media (SPOSM); Stips (public Q&A social media site); outreach replies by trained Sahar volunteers to suicidal posts; inductive thematic analysis of responses; real-world data.	The majority of responses expressed positive impact (70%); some reported barriers to engaging (16%); few reported negative impact (10%); findings suggest that outreach messages may foster help-seeking; findings highlight challenges such as emotional barriers and privacy concerns.
van der Burgt et al. (55)	Netherlands	Randomized controlled trial (RCT)	*n* = 772 (Website visitors)	Testing whether a brief barrier-reduction intervention (BRI) increases helpline contact among people with severe suicidal thoughts and low motivation.	113 Suicide Prevention website self-test; SIDAS screening; self-reported likelihood of contacting the helpline; satisfaction with the helpline.	No effect on the primary outcome (link usage: 33.1% BRI vs. 35.7% control); BRI increased the self-reported likelihood of later contacting the helpline and slightly increased satisfaction.
Educational (and counseling) measures						
Dreier et al. (35)	Germa6Gny, Austria, Switzerland	Mixed methods	*n* = 16 (Participants follow-up-interview)	Evaluating the unguided online program “8 Lives” to increase suicide literacy, reduce suicide stigma, and improve self-efficacy to seek support.	Unguided, browser-based online program, including lived-experience videos & evidence-based info, measures pre/post: suicide literacy, perceived stigma & self-stigma), support-seeking self-efficacy, distress, follow-up telephone interviews.	Qualitative follow-up interviews indicated enhanced processing of STB and increased help-seeking due to the program.
Frey et al. (37)	USA	RCT	*n* = 421 (Participants)	Examining a website for depression and suicidality in men with regard to suicidal ideation, depression, and risk and protective factors	Screening, offering of support services, and referral to the website (intervention group only)	Reduction in, among other outcomes, suicidal ideation; however, no differences were observed between the intervention and control groups.
van Spijker et al. (56)	Netherlands	RCT	*n* = 236 (Participants)	Testing the effectiveness of unguided online self-help to reduce suicidal thoughts.	Web-based self-help programm (6 modules).	A small but significant reduction in suicidal thoughts and worry; larger benefit for participants with repeated prior attempts.
Niederkrotenthaler et al. (57)	Austria	RCT	*n* = 266 (Participants)	Testing the effects of suicide awareness materials among adults with recent suicidal ideation or attempt.	Online exposure to 1) a layperson lived-experience article or 2) a mental health expert article vs. a control article; primary outcome: suicide risk; secondary outcomes: suicide-prevention knowledge and mood.	The lived-experience article showed immediate and 1-week sustained beneficial effects on suicide risk (small effect, stronger among those with a recent attempt) and increased suicide-prevention knowledge; no evidence of increased short-term risk.
Mishara et al. (58)	Canada	Quasi-experimental study	*n* = 131 (Family/Friends concerned about high-risk suicidal men)	Comparing the effects of four programs for family members/friends of high-risk suicidal men.	Four suicide prevention center programs (1) information session, (2) information plus follow-up, (3) rapid referral, and (4) telephone support; pre-, 2-month, and 6-month follow-up.	Reported reductions in men's suicidal ideation and suicide attempts and depressive symptoms; no difference between the programs.

Of the 24 studies, 29.2% (*n* = 7) examine the effects of text-based crisis intervention. This service was often linked to Lifelines that also enable contact via text (e.g., chats, forums, emails). All seven articles were published between 2017 and 2025. Sample sizes in these studies range from *n* = 106 to *n* = 39,911.

A total of 12.5% (*n* = 3) of the studies focus on outreach messages in various contexts. Outreach messages refer to proactive contact-in this case via text messages-with individuals who had been identified as at risk of developing STBs, based on their contributions online. The studies were published in 2019, 2022, and 2025. Two of these studies examine outreach messages in response to social media posts. One study examined outreach messages triggered by an online self-assessment tool. The sample sizes range from *n* = 116 to *n* = 1,403.

Five studies (20.8%) examined a web-based educational or informational intervention as well as a combination of informational intervention with counseling and referral to additional support services. The studies were published between 2005 and 2023, with sample sizes ranging from *n* = 16 to *n* = 421.

Of the 24 studies, six (25%) originated in Canada, five (20.8%) in the United States, and nine (37.5%) in Europe, of which five (20.8%) were conducted in the Netherlands. One study each (4.2%) was conducted in Israel, Taiwan, China, and Lebanon. All studies were published in English.

The results are organized thematically by type of intervention: voice-based crisis intervention (Lifelines), text-based crisis intervention (chats, emails), outreach messages in web-based contexts, and other educational/counseling measures.

### Voice-based crisis intervention (lifelines)

This measure accounts for the majority of the identified studies. A total of nine studies (37.5%) addresses the effects of voice-based crisis intervention for people with STBs via telephone (Lifelines). Counselors are typically available 24/7 or for extended hours via a phone number [e.g., (38); 18.5 h per day]. The Lifeline services have a very low access threshold, are generally free of charge, and are mostly anonymous.

The studies originate from different regions and therefore examine different Lifelines. Three of the nine studies (33.3%) examine the National Suicide Prevention Lifeline in the United States (36, 39, 40). Another study from the United States examined the Veteran Crisis Line (41). One study (11.1%) examines a Lifeline service in the United States and Canada (42), one examines the Samaritans' service in the United Kingdom (43), one the Taiwan National Suicide Prevention Hotline (44), and one Lebanon's National Emotional Support and Suicide Prevention Helpline (38).

All studies consistently demonstrate a reduction in suicidal distress following the use of telephone crisis intervention.

However, the outcome assessment varied considerably across studies and was grouped into three broad categories. First, several studies employed standardized instruments to assess suicidality or psychological distress before and after the conversation, including items from the Columbia Suicide Severity Rating Scale (C-SSRS) (38), the Mental State Rating Scale (MSRS), the Suicide Risk Scale (SRS) (44), and the Crisis Call Outcome Rating Scale (CCORS), which captures, among other things, suicidal urgency (45). Second, several studies assessed outcomes through callers' self-reports, either immediately after the conversation via a keypad system (42) or through a survey on a separate website (43). Third, several studies used the so-called silent monitoring method, in which trained individuals listened to the conversation and rated, among other things, the caller's level of distress (39, 45). Thus, while the operationalization of outcomes differed considerably across studies, the overall findings were generally consistent.

Across studies using self-reported outcomes, callers consistently reported feeling less suicidal, less lonely, less anxious, less depressed, and less overwhelmed after the conversation, while also reporting increased hope and feeling more supported, heard, and understood (39, 43). Studies using observer-rated or standardized measures likewise demonstrated reductions in suicidal distress, including lower CCORS scores following the intervention (45). One study by Gould et al. (40) examined the effects of one or more follow-up calls rather than the initial contact; 79.6% of participants reported that they believed the follow-up calls had prevented them from dying by suicide. Another study by Gould et al. (36) did not directly examine changes in STBs but instead assessed the uptake of recommended additional support services. Detailed results are presented in Table 2.

### Text-based crisis intervention

Seven studies (29.2%) address the effects of text-based crisis intervention. This is typically delivered via chats, email, or forums. There is a chat function or email address through which individuals can contact counselors and access support services. The majority of studies in this area also examine chat/email exchanges between counselors and users. However, one of the seven studies examines peer-to-peer interaction, that is, the moderated exchange between individuals with STBs in a forum associated with a website (46). Text-based crisis intervention is often offered alongside telephone crisis intervention. It generally provides an additional channel for accessing the support service. These services are typically comparable to telephone in terms of low access thresholds. They are usually free of charge and anonymous. As with voice-based crisis interventions, emergency services are typically contacted only in cases where there is an imminent or ongoing risk of harm to self or others, and when a less invasive safety plan for the caller or texter cannot be collaboratively developed with the individual.

All studies in this intervention area originate from only three regions, unlike the voice-based studies described above: from Canada/Quebec via the Canada Suicide Prevention Service (CSPS) or the website suicide.ca (47–49), from the United States via the National Suicide Prevention Lifeline Crisis Chat (50), and from the Netherlands via the 113 Online Chat (46, 51, 52). The analytical methods were somewhat more uniform for this measure type than for voice-based crisis interventions. Most studies analyzed the transcripts of interactions concerning STB-related distress, among other aspects (47, 49, 52), and employed participant surveys and/or pre- and post-questionnaires (e.g., SIDAS) as well as standardized counsellor reports (48–51).

Across studies evaluating counselor-delivered text-based crisis intervention, findings were highly consistent, comparable to those of telephone counseling (47, 52) and consistently demonstrated a reduction in STB-related distress. In the survey by Cote et al. (48), 82.6% reported feeling better after the chat, 76.8% described greater control over suicidal thoughts, and 72.5% felt more capable of coping with problems. In the survey by Gould et al. (50), 45% of users reported feeling less suicidal. Janssen et al. (51) also found that participants reported improvements in suicidal ideation, psychological distress, hopelessness, feelings of alienation, perceived burdensomeness, and sense of belonging. The results from Mokkenstorm et al. (52) likewise demonstrate positive effects of chat-based crisis intervention on suicidal distress and improvement in emotional states.

In contrast to the studies evaluating counsellor-delivered text-based crisis intervention, Mokkenstorm et al. (46) examined a moderated peer-to-peer forum and found a more mixed pattern of effects. Positive effects are noted here as well: 35% of the users with STBs reported feeling better, users positively valued the peer support, described feelings of acceptance and understanding, and appreciated anonymity. At the same time, the study also identified potentially harmful effects. Twelve percent reported feeling worse after using the forum, 12–13% reported searching for suicide methods, and 22% reported distress caused by posts from other users.

### Outreach messages in web-based contexts

Of the 24 studies, three (12.5%) fall into this intervention area. These measures involve identifying people with STBs in web-based environments (e.g., websites, forums, social media) and proactively contacting them via the relevant platform. The three studies evaluated different outreach approaches. Two studies identified suicide-related online content and subsequently offered anonymous chat-based crisis intervention (53, 54), whereas one study reached out to individuals following an online self-assessment by providing personalized messages addressing barriers to help-seeking along with a direct link to Lifeline (55).

Overall, proactive outreach approaches yielded promising findings. Liu et al. (53) showed that machine learning could identify individuals with STBs with adequate accuracy and that participants who engaged in the intervention subsequently used more future-oriented and fewer death-related words during chat exchanges. Similarly, Keasar et al. (54) reported that 69.8% of responses to proactive outreach were positive, with users describing feelings of hope and being understood.

In contrast, van der Burgt et al. (55) found that personalized outreach following an online self-assessment did not significantly increase uptake of Lifeline services compared with a standardized counseling message.

### Educational (and counseling) measures

Five of the 24 studies (20.8%) were categorized as educational (and counseling) measures. These studies evaluated various types of interventions and employed different study designs, including randomized controlled trials (RCTs), qualitative evaluations, and third-party assessments.

In a qualitative evaluation, Dreier et al. (35) examined an online educational program containing information about STBs, available support services, and experiential accounts from individuals with STBs. The study was conducted primarily with participants from Germany, as well as from Austria and Switzerland. The qualitative evaluation included 16 participants.

Fifty percent of participants were individuals with STBs. Participants reported that the program helped them process STBs and motivated them to seek professional help.

Three studies employed controlled designs to evaluate the effects of web-based educational interventions. Frey et al. (37) evaluated a web-based intervention providing information and support services on depression and suicide tailored specifically to men. Using a randomized controlled design, the study compared reductions in suicidal ideation between the intervention group and a control group. Both groups received a screening for depression and suicide risk as well as information on available support services. Only the intervention group was additionally directed to the intervention website. Both groups showed a reduction in suicidal ideation over time. However, no statistically significant differences were found between the intervention and control groups.

In another randomized controlled trial, van Spijker et al. (56) evaluated a six-module web-based self-help program and demonstrated that suicidal ideation was significantly reduced in the intervention group compared to the waitlist control group. Niederkrotenthaler et al. (57) used an experimental controlled design and found a reduction in suicide risk compared with the control group after exposure to suicide-related texts. Overall, controlled studies suggest potential benefits of web-based educational interventions, although findings are heterogeneous, with one RCT showing no significant differences between intervention and control groups.

A distinct approach was evaluated by Mishara et al. (58) in Canada, where the intervention targeted third parties rather than individuals with STBs themselves. Callers to Lifeline who were concerned about a man affected by STBs were offered one of four educational/counseling measures: 1. one information session, 2. option 1 plus telephone follow-up, 3. rapid referral to a specialized treatment service for STBs, and 4. longer-term telephone counseling for the third party. A significant decrease in STBs was found, along with a lower likelihood of a suicide attempt at 2 months and less intense suicidal ideation among the affected individuals. However, these findings should be interpreted with caution because outcomes related to STBs were assessed through third-party proxy reports rather than by the individuals experiencing suicidal thoughts themselves, which may introduce reporting bias. Furthermore, the intervention included counseling directed at third parties, making it difficult to attribute effects solely to the educational component. Thus, while the study suggests potential benefits of involving third parties in suicide prevention, the interpretation of effects is limited by the indirect assessment of outcomes.

## Discussion

This scoping review provides a systematic overview of the existing evidence regarding on secondary and tertiary suicide prevention measures for adults. In summary, robust evidence exists for voice- and text-based crisis interventions provided through suicide-related lifelines. However, several gaps remain, particularly with regard to the evidence for alternative forms of care or measures tailored to specific target populations.

A large proportion of the studies included in this review on the effects of secondary and tertiary suicide prevention measures on STBs among adults examined voiced- and text-based crisis interventions (16 of 24; 66.7%). Most of these interventions are embedded within so-called lifeline services. Individuals experiencing STBs can typically access counselors via telephone or chat 24/7, free of charge and anonymously. Overall, the findings present a consistent picture: both language- and text-based crisis interventions tend to achieve the intended effects. They reduce STBs [e.g., (41, 43, 49)], alleviate distress, and have a positive impact on wellbeing [e.g., (42, 51)]. Research on language-based crisis interventions has a longer tradition, whereas studies on text-based crisis interventions have emerged primarily in recent years. Evidence suggests that text-based crisis interventions can achieve outcomes comparable to those of language-based services (52). Yet, one study examining moderated peer-to-peer text communication also identified potential risks (46).

The remaining eight studies provide insight into two additional areas of potentially effective secondary and tertiary suicide prevention. One area encompasses web-based outreach messages directed at individuals at risk of suicide. Emerging technologies, including artificial intelligence (AI), offer new possibilities for identifying individuals at risk in online communication (e.g., via social media posts) and proactively engaging with them (53–55). The three identified studies on outreach messages show heterogeneous findings. The interventions they examined had been implemented in diverse contexts and were evaluated using different methodological approaches. However, overall, the findings outline the potential of web-based outreach, which warrants further investigation. At the same time, existing research reports barriers and unresolved issues, including concerns related to data protection and privacy. The third thematic area identified in our review covers informational and training programs. These interventions also vary considerably in their specific design. Reported effects tend to be modest (35, 37, 56); nevertheless, all findings indicate a potential for reducing STBs, particularly when programs incorporate reports of people with lived experience (35, 57).

The findings presented as part of this scoping review largely align with the existing literature that describes lifeline services as effective interventions for reducing STBs [e.g., (16)]. The effectiveness of text-based crisis interventions integrates well into this body of evidence, as these services are often delivered by the same organizations and are likely to involve similarly trained counselors. The lifelines (voice- and text-based) are generally suicide-specific services.

Web-based outreach messages are a relatively recent phenomenon, have been accordingly studied to a much lesser extent to date, and are closely linked to current technological developments. After at-risk individuals have been identified and approached, they are ultimately offered a text-based crisis intervention. The findings regarding outreach messages point in a similar direction as these.

Rassy et al. (13) reported promising findings regarding the identification of at-risk individuals through web-based solutions. Ethical and safety-related concerns highlighted in the included studies are reflected in this context. Rassy et al. (13) further emphasized the challenges arising from the heterogeneity of the approaches used and the varying levels of evidence for web-based and ICT-supported (information and communication technology) suicide prevention strategies. The review additionally points to positive effects of self-management programs, which are consistent with our findings. Evidence on the effects of training programs enhancing knowledge about suicidality and increasing the willingness to seek help is also reported by Ries et al. (59) and King et al. (60). Positive effects of training programs on knowledge and self-efficacy among gatekeepers have also been documented by Liu et al. (61). Although gatekeeper training was excluded from this review, existing relevant evidence still confirms its positive value.

Several limitations must be noted. Most included studies assessed short-term rather than long-term effects. Apart from a few brief follow-up periods, outcomes were typically measured immediately after the intervention. Only three studies included referral to further support services as an outcome (36, 55, 58). Evidence on medium and long-term effects is lacking so far and further research should focus on the stability of the effects.

Face-to-face crisis interventions and counseling services conducted outside of medical or psychotherapeutic settings were not addressed by any of the studies. In Germany, a wide range of such counseling services exists (5). To our knowledge these services have not been subject of empirical evaluation with regard to their effectiveness. It thus remains unknown how and for whom these services work. It is conceivable, however, that such services are less prevalent in other regions of the world, which may explain the lack of studies in this field. Future research should address this gap.

Only three of the 24 included studies (12.5%), (37, 58), (41), examined specific groups with an elevated risk (men, veterans), while other important target groups (e.g., older adults or queer individuals) were not represented. Similarly, reviews focusing on particular target groups [e.g., (62)] did not identify studies suitable for inclusion in this review. Future research on the effects of secondary and tertiary suicide prevention measures on SIB should therefore place greater emphasis on investigating specific high-risk populations.

Another important limitation is that the majority of the included studies were observational in design, with only a small number of (randomized) controlled trials (RCTs). Observational studies do not permit causal inferences. The scarcity of RCTs may be attributable to the practical and ethical challenges inherent in conducting RCTs in the context of secondary and tertiary suicide prevention. Nonetheless, this issue should be addressed in future research. In particular, newer web-based and ICT-supported interventions-an increasingly diverse and rapidly evolving field-should be accompanied by rigorous evaluations. The development and adherence to scientific standards, as well as careful consideration of safety and ethical aspects, are especially critical in this domain. One potential limitation of this scoping review is the regional bias introduced by including only articles published in English and German. A key strength of the review is its focus on evidence-based studies; however, methodological quality was heterogeneous, and only a small number of randomized controlled trials (RCTs) were identified.

A further limitation is the restriction of the search period to studies published from 2005 onwards, which may have resulted in the omission of earlier evidence on low-threshold suicide prevention measures.

In summary, the findings support the implementation of low-threshold, language- or text-based crisis interventions within lifeline services and suggest that informational and training programs, as well as outreach messages, may represent valuable complementary tools. A potential future application of AI-based approaches may be the identification of suicide-++related content in online environments to enable proactive outreach. Such approaches may provide new opportunities to engage individuals with STBs who are not currently connected to support services, although implementation requires careful consideration of ethical and privacy challenges. With the exception of studies on voice-based crisis interventions and one study on training programs (58), most included studies have been published in recent years. This trend may indicate emerging opportunities for innovative low-threshold secondary and tertiary suicide prevention measures.

## Data Availability

Publicly available datasets were analyzed in this study. This data can be found here: https://osf.io/kjt39. Further inquiries can be directed to the corresponding author.
