# Supplementary material for: Low-threshold secondary and tertiary suicide prevention measures for individuals with suicid-related thoughts and behaviors: a scoping review
Source: Front Public Health. 2026 Sep 28;14:1868906. doi: 10.3389/fpubh.2026.1868906 (PMC13655041; doi:10.3389/fpubh.2026.1868906)
Supplement: Supplementary file 1 [file Supplementary_File_1.docx]

**Supplementary Material: Searchstrings**

Pubmed (https://pubmed.ncbi.nlm.nih.gov/)

(

(suicid*[ti] AND prevention*[ti])

AND

(suicid*[ab] AND prevention*[ab])

)

AND

(

"Suicide Prevention"[Mesh] OR

"Crisis Intervention"[Mesh] OR

"Aftercare"[Mesh] OR

"Health Education"[Mesh] OR

"Health Promotion"[Mesh] OR

"Hotlines"[Mesh] OR

hotline*[tiab] OR

helpline*[tiab] OR

"Counseling"[Mesh] OR

counsel*[tiab] OR

"Peer Group"[Mesh] OR

"peer support"[tiab] OR

psychoeducation*[tiab] OR

"information campaign*"[tiab] OR

"Social Media"[Mesh] OR

"social media campaign*"[tiab] OR

app[tiab] OR

apps[tiab] OR

"online resource*"[tiab] OR

e-learning[tiab] OR

gatekeeper*[tiab] OR

"Bereavement"[Mesh] OR

"bereaved by suicide"[tiab]

)

AND

(

"Health Services Accessibility"[Mesh] OR accessibility[tiab] OR

"Community-Institutional Relations"[Mesh] OR outreach[tiab] OR "community outreach"[tiab] OR

"Health Services"[Mesh] AND (utilization[tiab] OR "health service utilization"[tiab]) OR

"Health Services Accessibility"[Mesh] AND (barriers[tiab] OR "health care barrier*"[tiab]) OR

"Health Knowledge, Attitudes, Practice"[Mesh] OR "help seeking"[tiab] OR "low-threshold"[tiab]

Filters applied: Classical Article, Clinical Study, Clinical Trial, Meta-Analysis, Multicenter Study, Network Meta-Analysis, Randomized Controlled Trial, Review, Scoping Review, Systematic Review, English, German, Humans, from 2005/1/1 - 2025/9/1

Web of Sciences (https://www.webofscience.com/wos/woscc/smart-search)

(TI=suicid* AND TI=prevention*)

AND

(TOPIC=suicid* AND TOPIC=prevention*)

AND

(

AB="suicide prevention" OR AB="crisis intervention" OR AB=aftercare OR

AB="health education" OR AB="health promotion" OR

AB=hotline* OR AB=helpline* OR AB=counseling OR AB="peer support" OR

AB=psychoeducation* OR AB="information campaign*" OR

AB="social media" OR AB="social media campaign*" OR AB=app OR AB=apps OR

AB="online resource*" OR AB=e-learning OR AB=gatekeeper* OR

AB="bereaved by suicide" OR AB=bereaved

)

AND

(

AB="health services accessibility" OR AB=accessibility OR

AB=outreach OR AB="community outreach" OR

AB="health service utilization" OR AB=utilization OR

AB=barrier* OR AB="health care barrier*" OR

AB="help seeking" OR AB="low-threshold"

)

Additional criteria: January 1, 2005 – September 1, 2025; languages: English and German
